# How common are complications following polypropylene mesh, biological xenograft and native tissue surgery for pelvic organ prolapse? A secondary analysis from the PROSPECT trial

**DOI:** 10.1111/1471-0528.16897

**Published:** 2021-09-27

**Authors:** FM Reid, A Elders, S Breeman, RM Freeman, the PROSPECT study group, CMA Glazener, C Hemming, KG Cooper, ARB Smith, S Hagen, I Montgomery, M Kilonzo, D Boyers, A McDonald, G McPherson, G MacLennan, J Norrie

**Affiliations:** ^1^ The Warrell Unit Saint Mary’s Hospital Manchester University NHS Foundation Trust Manchester Academic Health Science Centre Manchester UK; ^2^ Faculty of Medical & Human Sciences Manchester Academic Health Science Centre Institute of Human Development University of Manchester Manchester UK; ^3^ NMAHP Research Unit Glasgow Caledonian University Glasgow UK; ^4^ Health Services Research Unit Centre for Healthcare Randomised Trial University of Aberdeen Aberdeen UK; ^5^ Department of Obstetrics and Gynaecology Plymouth Hospitals NHS Trust Plymouth UK

**Keywords:** Cumberlege report, dyspareunia, IUGA/ICS complications classification, pain, polypropylene mesh, prolapse, surgery, surgical complications

## Abstract

**Objective:**

To report complication rates following prolapse surgery using polypropylene mesh inlay, polypropylene mesh kit, biological collagen xenografts and native tissue repairs.

**Design:**

Secondary analysis of the PROSPECT randomised controlled trial and cohort study.

**Setting:**

Thirty‐five UK hospitals.

**Population:**

A total of 2632 women undergoing anterior and/or posterior vaginal prolapse repair.

**Methods:**

Event rates were calculated for all complications. Analysis was by treatment received.

**Main outcome measures:**

IUGA/ICS classification of complications and validated patient reported outcome measures.

**Results:**

At baseline, 8.4% of women had ‘generic’ pain/discomfort; at 2 years following surgery, there was an improvement in all four groups; however, 3.0% of women developed de novo extreme generic pain. At 24 months de novo vaginal tightness occurred in 1.6% of native tissue, 1.2% of biological xenograft, 0.3% of mesh inlay and 3.6% of mesh kit. Severe dyspareunia occurred in 4.8% of native tissue, 4.2% of biological xenograft, 3.4% of mesh inlay repairs and 13.0% of mesh kits. De novo severe dyspareunia occurred in 3.5% of native tissue, 3.5% of biological xenograft, 1.4% of mesh inlays and 4.8% of mesh kits. Complications requiring re‐admission to hospital, unrelated to mesh, affected 1 in 24 women; the most common reasons for re‐admission were vaginal adhesions, urinary retention, infection and constipation.

**Conclusions:**

This is the first study to address the complications of vaginal mesh used for prolapse surgery alongside data from both native tissue and biological xenograft. It demonstrates the complexity of assessing pain and that all types of prolapse surgery have low surgical morbidity and a low rate of severe complications.

**Tweetable abstract:**

A prospective study of 2362 women undergoing vaginal mesh, xenograft or native tissue repair found low surgical morbidity and low rates of severe complications.

## Tweetable abstract

A prospective study of 2362 women undergoing vaginal mesh, xenograft or native tissue repair found low surgical morbidity and low rates of severe complications.

## Introduction

Transvaginal polypropylene mesh for pelvic organ prolapse (POP) and stress urinary incontinence has been the subject of much controversy with its use being suspended in some countries as the result of complications,^1^ and several series including the management of such complications have been published.^2^, ^3^

The evidence collected during the Cumberlege inquiry in the UK^4^ has highlighted serious side effects of the use of mesh and short comings in medical practice. The report, *First do no harm*, states that there is a risk of harm not only from the primary surgical procedure but also from subsequent mesh removal surgery.

Very few studies have compared the complications of POP surgery using polypropylene mesh or biological xenografts compared with native tissue surgery.

PROSPECT, one of the largest multicentre comprehensive cohort studies with an embedded randomised controlled trial comparing native tissue, biological xenograft and mesh for transvaginal repair of anterior and posterior compartment pelvic organ prolapse, revealed no significant differences in patient‐reported outcomes at 2 years.^5^, ^6^, ^7^ Serious adverse effects, defined as causing death, requiring admission to hospital or prolongation of existing hospital admission, resulting in significant incapacity or disability, or otherwise considered important by the investigator were comparable across treatment groups.

To provide more detailed information about mesh‐related complications, especially pain, a secondary analysis of the complete PROSPECT data set, both randomised and those in the comprehensive cohort study, was undertaken using the internationally recommended International Urogynecological Association (IUGA)/International Continence Society (ICS) classification.^8^, ^9^ This provides information about the type of complication, the anatomical site where it occurred, severity and the time of onset. There is also a subclassification of pain severity, from asymptomatic to unprovoked spontaneous pain. Studies have shown that following instruction and training there is good inter‐observer reliability^10^ and others report that this is a useful method for assessing the severity of complications.^2^, ^3^ Unlike PROSPECT, most studies have been retrospective and have not included a control group of women undergoing native tissue surgery.

Following the Cumberlege Report, the aim of this PROSPECT secondary analysis is to provide more detailed information, using validated patient‐reported outcome measures (PROMS) and a standardised complication classification system, to help in patient counselling.

## Methods

### Participants

All participants recruited into the PROSPECT study who underwent a transvaginal anterior and/or posterior prolapse operation (either primary or secondary repair) were included in this secondary analysis. The study group included a lay person who was involved in all aspects of the study, from planning to reporting. Randomised and non‐randomised women having a PROSPECT study treatment for either a primary repair or repeat procedure were included and the operation could include concomitant uterine, vault or continence surgery. Exclusion criteria were concomitant abdominal surgery (continence procedures, hysterectomy, uterine suspension or vault fixation) or upper compartment prolapse only. All women provided written informed consent to participate in the PROSPECT study.

### Procedure

Participating surgeons used their usual surgical techniques for native tissue repairs, polypropylene mesh inlay, biological xenograft and mesh kits. The mesh was non‐absorbable type 1 monofilament microporous polypropylene mesh, with or without absorbable coating (‘hybrid mesh’). Mesh kits were defined as those that used removable trocars to place the mesh. Bespoke or pre‐cut ‘armed’ mesh was classed as an inlay if trocars were not used. The biological xenografts were porcine acellular collagen matrix, porcine small intestine submucosa or bovine dermal grafts. Mesh inlays and biological xenografts were inserted below the fascial layer if possible and secured with peripheral sutures to the fascia or arcus tendinous fascia pelvis (‘white line’) or the sacrospinous ligament.

### Outcomes

Information regarding the surgical procedure and any complications before discharge was collected directly from the research team at each participating hospital. Information on study‐related adverse events, self‐reported pain and need for readmission/further treatment was collected by participant‐completed postal questionnaires at 6 months, 1 and 2 years after surgery. Reports from participants were verified by the local research team where possible.

Generic pain/discomfort was recorded from the pain questions within EQ‐5D‐3L, the questionnaire has three responses for pain/discomfort (none, moderate or severe)^11^ Serious adverse events were subsequently defined using the recommended IUGA/ICS classifications, including the subclassification for pain.^8^, ^9^

There are currently no agreed Core Outcomes in Women's and Newborn Health (CROWN) for pelvic floor dysfunction. A panel of validated core outcomes relevant to women’s symptoms was completed by postal questionnaire. These included the Pelvic Organ Prolapse Symptom Score (POP‐SS),^12^ prolapse‐specific quality‐of‐life and generic quality‐of‐life based on the EQ‐5D‐3L.^11^ Bladder, bowel and sexual function were measured using validated or adapted International Consultation on Incontinence Questionnaires (ICIQ).^13^ Several individual questions were reported from these validated questionnaires.

Objective measurement of prolapse stage used the POP‐Q system^14^ at 1 year after surgery (randomised women only).

### Statistical analysis

Analysis was carried out by treatment received according to study treatment (native tissue repair, mesh inlay, biological xenograft, or mesh kit). Baseline characteristics and complication rates are presented for each group and for all participants combined. Clopper–Pearson exact 95% CI are reported. Complication rates for each category presented are calculated as the percentage of participants who experienced one or more events in that category. Only observed data are included in the analysis, e.g. de novo rates were calculated only for participants with data collected at baseline and at follow up. The analysis sought to describe complications for different surgical procedures rather than to examine any hypotheses and hence no inferential tests were conducted to compare treatments. Analysis was conducted using SAS v 9.4 (SAS Institute, Cary, NC, USA).

## Results

The PROSPECT study operated on 2632 women included in this analysis; 1712 had a native tissue repair, 482 received a polypropylene mesh inlay, 360 a biological xenograft and 78 a mesh kit. The baseline characteristics by treatment received are shown, for each group, in Table 1. Of note within the randomised controlled trial section of PROSPECT biological xenograft was not used for recurrent prolapse cases and mesh kits were not used for primary cases.

**Table 1:** Baseline characteristics by treatment received

				All procedures			Native tissue repair			Mesh inlay			Biological xenograft			Mesh kit		
Age (years)	*N*	Mean	(SD)	2632	59.9	(10.7)	1712	60.0	(10.9)	482	60.0	(10.1)	360	58.6	(10.1)	78	63.2	(10.0)
Parity	*N*	Median	(Range)	2622	2	(0–12)	1702	2	(0–12)	482	2	(0–9)	360	2	(0–7)	78	2	(1–7)
Prolapse symptoms																		
POP‐SS	*N*	Mean	(SD)	2434	13.8	(5.8)	1568	13.5	(5.8)	456	14.0	(5.7)	336	14.1	(5.7)	74	15.5	(5.9)
Symptomatic prolapse	*N*	n	(%)	2434	2422	(99.5)	1568	1559	(99.4%)	456	454	(99.6%)	336	336	(100%)	74	73	(98.6%)
Prolapse‐related QoL	*N*	Mean	(SD)	2391	6.7	(2.7)	1536	6.6	(2.7)	450	6.7	(2.6)	331	6.7	(2.7)	74	7.3	(2.2)
EQ‐5D‐3L	*N*	n	(%)	2361	0.70	(0.25)	1512	0.70	(0.25)	450	0.71	(0.23)	326	0.70	(0.26)	73	0.65	(0.25)
Severe UI	*N*	n	(%)	2397	507	(21.2%)	1536	318	(20.7%)	453	100	(22.1%)	334	75	(22.5%)	74	14	(18.9%)
Faecal incontinence (any)	*N*	n	(%)	2405	841	(35.0%)	1551	525	(33.8%)	449	166	(37.0%)	333	123	(36.9%)	72	27	(37.5%)
ICI Vaginal Symptoms Score	*N*	Mean	(SD)	2159	22.5	(9.2)	1383	22.1	(9.0)	407	22.9	(9.8)	306	23.2	(9.3)	63	23.3	(9.4)
Previous surgery																		
Prolapse repair	*N*	*n*	(%)	2632	609	(23.1%)	1712	339	(19.8%)	482	155	(32.2%)	360	49	(13.6%)	78	66	(84.6%)
Vault repair	*N*	*n*	(%)	2632	92	(3.5%)	1712	43	(2.5%)	482	28	(5.8%)	360	9	(2.5%)	78	12	(15.4%)
Hysterectomy	*N*	*n*	(%)	2631	884	(33.6%)	1711	510	(29.8%)	482	201	(41.7%)	360	127	(35.3%)	78	46	(59.0%)
Surgery for urinary incontinence	*N*	*n*	(%)	349	53	(15.2%)	181	20	(11.0%)	94	18	(19.1%)	19	3	(15.8%)	55	12	(21.8%)
Overall POP‐Q stage																		
Stage 0	*N*	*n*	(%)	2465	3	(0.1%)	1567	2	(0.1%)	471	0	(0.0%)	354	1	(0.3%)	73	0	(0.0%)
Stage 1	*N*	*n*	(%)	2465	27	(1.1%)	1567	18	(1.1%)	471	5	(1.1%)	354	3	(0.8%)	73	1	(1.4%)
Stage 2	*N*	*n*	(%)	2465	1492	(60.5%)	1567	976	(62.3%)	471	271	(57.5%)	354	203	(57.3%)	73	42	(57.5%)
Stage 3	*N*	*n*	(%)	2465	893	(36.2%)	1567	535	(34.1%)	471	189	(40.1%)	354	141	(39.8%)	73	28	(38.4%)
Stage 4	*N*	*n*	(%)	2465	50	(2.0%)	1567	36	(2.3%)	471	6	(1.3%)	354	6	(1.7%)	73	2	(2.7%)
Leading edge >0 cm	*N*	*n*	(%)	2275	1429	(62.8%)	1425	858	(60.2%)	443	303	(68.4%)	341	224	(65.7%)	66	44	(66.7%)

Table 2 summarises the complications of prolapse surgery and demonstrates that the perioperative safety of all types of prolapse surgery is good. A bladder injury was sustained by 1 in 263 women (0.4%, 95% CI 0.2–0.7%) and bowel injury by 1 in 526 (0.2%, 95% CI 0.1–0.4%, Table S1).

**Table 2:** Summary of complications from the PROSPECT study

				All procedures *N* = 2632			Native tissue repair *N* = 1712			Mesh inlay *N* = 482			Biological xenograft *N* = 360			Mesh kit *N* = 78		
Intraoperative complications																		
Injury to organs		*n*	(%)		13	(0.5%)		10	(0.6%)		2	(0.4%)		1	(0.3%)		0	(0.0%)
Excess blood loss		*n*	(%)		16	(0.6%)		9	(0.5%)		4	(0.8%)		3	(0.8%)		0	(0.0%)
Postoperative complications																		
Return to theatre <72 hours		*n*	(%)		19	(0.7%)		11	(0.6%)		5	(1.0%)		2	(0.6%)		1	(1.3%)
Catheterisation required >10 days		*n*	(%)		103	(3.9%)		70	(4.1%)		14	(2.9%)		12	(3.3%)		7	(9.0%)
Complications within 24 months																		
Urinary retention		*n*	(%)		78	(3.0%)		53	(3.1%)		12	(2.5%)		9	(2.5%)		4	(5.1%)
Vaginal adhesions		*n*	(%)		44	(1.7%)		25	(1.5%)		6	(1.2%)		12	(3.3%)		1	(1.3%)
Resulting in hospitalisation		*n*	(%)		152	(5.8%)		70	(4.1%)		48	(10.0%)		29	(8.1%)		5	(6.4%)
Related to mesh		*n*	(%)		40	(1.5%)		2	(0.1%)		34	(7.1%)		1	(0.3%)		3	(3.8%)
Unrelated to mesh		*n*	(%)		112	(4.3%)		68	(4.0%)		14	(2.9%)		28	(7.8%)		2	(2.6%)
Patient compromise*		*n*	(%)		67	(2.5%)		39	(2.3%)		15	(3.1%)		10	(2.8%)		3	(3.8%)
Mesh complications** resulting in surgery within 24 months (from questionnaire data)																		
Surgical removal of mesh	*N*	*n*	(%)	2074	38	(1.8%)	1329	2	(0.2%)	390	31	(7.9%)	290	0	(0.0%)	65	5	(7.7%)
Repeat prolapse surgery	*N*	*n*	(%)	2075	7	(0.3%)	1329	0	(0.0%)	391	7	(1.8%)	290	0	(0.0%)	65	0	(0.0%)
De novo urinary incontinence (from questionnaire data)																		
At 12 months	*N*	*n*	(%)	2404	151	(6.3%)	1548	95	(6.1%)	447	28	(6.3%)	336	24	(7.1%)	73	4	(5.5%)
At 24 months	*N*	*n*	(%)	2387	149	(6.2%)	1542	94	(6.1%)	442	30	(6.8%)	331	23	(6.9%)	72	2	(2.8%)

*IUGA Classification 7 A, B and C (Although there were no deaths).

**Includes complications resulting from concomitant procedures in which mesh may have been used.

Excessive blood loss, clinically estimated as greater than 500 ml, was reported in 1 in 165 cases (0.6%, 95% CI 0.3–1.0%); there were none in the mesh kit group.

Return to theatre within the first 72 hours occurred in 1 in 139 cases (0.7%, 95% CI 0.4–1.1%), the majority of these were for haemorrhage.

Prolonged catheterisation (more than 10 days) occurred in 3.9% (95% CI 3.2–4.7%) of all cases, although it was more common in the mesh kit group.

At 24 months, de novo urinary incontinence, of any type, occurred in approximately 1 in 16 of all cases (6.2%, 95% CI 5.3–7.3%) with the exception of the mesh kit group, in which it was 1 in 36 cases (2.8%, 95% CI 0.3–9.7%).

Postoperative vaginal adhesions were found in 1 in 60 women (1.7%, 95% CI 1.2–2.2%) and were most common following a xenograft (1 in 30, 3.3%, 95% CI 1.7–5.8%).

Re‐admission to hospital for complications of surgery over the 2‐year period, unrelated to mesh, was relatively common affecting 1 in 24 women (4.3%, 95% CI 3.5–5.1%); the most common reasons for re‐admission were for management of vaginal adhesions, urinary retention, postoperative infection and constipation.

Despite the routine use of perioperative prophylactic antibiotics, infection was not uncommon, affecting approximately 1 in 40 women (2.4%, 95% CI 1.9–3.1%) although severe infection associated with abscess was rare, affecting fewer than 1 in 1000 women (0.1%, 95% CI 0.01–0.3%). There was only one case of pulmonary embolism reported following surgery.

In those who received mesh, surgical removal of mesh occurred in 7.9% (95% CI 5.5–11.1%) of cases, although the exact extent of removal was not recorded. Mesh exposure was found in 12.0% of women; however, 64.2% of these exposures were asymptomatic (Table S1).

The IUGA/ICS classification of complications is shown in Table 3. The most severe injuries were in Category 7 ‘patient compromise’, which is divided into three subcategories (Table S1). Category 7A (bleeding complication including haematoma) was common, 1 in 46 cases overall (2.2%, 95% CI 1.6–2.8%). However, need for major resuscitation or intensive care was uncommon, 1 in 239 cases (0.4%, 95% CI 0.2–0.7%) and there were no deaths (7C) related to surgery.

**Table 3:** IUGA classifications of complications related to prolapse repairs

			All procedures *N* = 2632		Native tissue repair *N* = 1712		Mesh inlay *N* = 482		Biological xenograft *N* = 360		Mesh kit *N* = 78	
General description												
1. Vaginal: no epithelial separation	*n*	(%)	178	(6.8%)	104	(6.1%)	30	(6.2%)	37	(10.3%)	7	(9.0%)
2. Vaginal: smaller, ≤1 cm exposure	*n*	(%)	49	(1.9%)	3	(0.2%)	39	(8.1%)	1	(0.3%)	6	(7.7%)
3. Vaginal: larger, >1 cm exposure, or any extrusion	*n*	(%)	24	(0.9%)	2	(0.1%)	21	(4.4%)	0	(0.0%)	1	(1.3%)
4. Urinary tract: compromise or perforation	*n*	(%)	84	(3.2%)	57	(3.3%)	13	(2.7%)	10	(2.8%)	4	(5.1%)
5. Rectal or bowel: compromise or perforation	*n*	(%)	5	(0.2%)	1	(0.1%)	3	(0.6%)	1	(0.3%)	0	(0.0%)
6. Skin or musculoskeletal: complications	*n*	(%)	15	(0.6%)	8	(0.5%)	6	(1.2%)	1	(0.3%)	0	(0.0%)
7. Patient: compromise*	*n*	(%)	67	(2.5%)	39	(2.3%)	15	(3.1%)	10	(2.8%)	3	(3.8%)
Time (clinically diagnosed)												
T1: Intraoperative to 48 hours	*n*	(%)	9	(0.3%)	7	(0.4%)	2	(0.4%)	0	(0.0%)	0	(0.0%)
T2: 48 hours to 2 months	*n*	(%)	7	(0.3%)	6	(0.4%)	0	(0.0%)	1	(0.3%)	0	(0.0%)
T3: 2–12 months	*n*	(%)	316	(12.0%)	172	(10.0%)	85	(17.6%)	46	(12.8%)	13	(16.7%)
T4: Over 12 months	*n*	(%)	63	(2.4%)	18	(1.1%)	30	(6.2%)	11	(3.1%)	4	(5.1%)
Site												
S1: Vaginal: area of suture line	*n*	(%)	104	(4.0%)	34	(2.0%)	48	(10.0%)	19	(5.3%)	3	(3.8%)
S2: Vaginal: away from suture line	*n*	(%)	168	(6.4%)	88	(5.1%)	43	(8.9%)	25	(6.9%)	12	(15.4%)
S3: Adjoining viscus/trocar passage**	*n*	(%)	98	(3.7%)	66	(3.9%)	17	(3.5%)	11	(3.1%)	4	(5.1%)
S4: Other skin or musculoskeletal site	*n*	(%)	17	(0.6%)	10	(0.6%)	6	(1.2%)	1	(0.3%)	0	(0.0%)
S5: Intra‐abdominal	*n*	(%)	17	(0.6%)	12	(0.7%)	3	(0.6%)	2	(0.6%)	0	(0.0%)
Pain												
a: Asymptomatic or no pain	*n*	(%)	144	(5.5%)	72	(4.2%)	46	(9.5%)	21	(5.8%)	5	(6.4%)
b: Provoked pain only	*n*	(%)	8	(0.3%)	3	(0.2%)	2	(0.4%)	2	(0.6%)	1	(1.3%)
c: Pain during sexual intercourse	*n*	(%)	33	(1.3%)	20	(1.2%)	8	(1.7%)	5	(1.4%)	0	(0.0%)
d: Pain during physical activities	*n*	(%)	4	(0.2%)	3	(0.2%)	1	(0.2%)	0	(0.0%)	0	(0.0%)
e: Spontaneous pain	*n*	(%)	65	(2.5%)	34	(2.0%)	22	(4.6%)	7	(1.9%)	2	(2.6%)
Unspecified	*n*	(%)	131	(5.0%)	67	(3.9%)	35	(7.3%)	21	(5.8%)	8	(10.3%)

*Patient compromise 7A: Bleeding complication including haematoma; 7B: Major degree of resuscitation or intensive care; 7C: Mortality.

**Adjoining viscus (e.g. bladder or bowel) for native tissue repairs and trocar passage for mesh repairs.

Overall repeat prolapse surgery in the first 2 years was uncommon – only 0.3% (95% CI 0.1–0.7%) and all occurred in the mesh inlay group.

Of note there was little difference in complication rates including pain, between primary and secondary surgery for standard native tissue repairs and mesh inlays (Table S2).

Table 4, Table S3 and the radar plots (Figure S1) provide further details of the pain associated with all types of prolapse surgery.

**Table 4:** Rates of self‐reported pain

				All procedures			Native tissue repair			Mesh inlay			Biological xenograft			Mesh kit		
Generic pain (‘extreme pain or discomfort’)																		
Baseline	*N*	*n*	(%)	2400	201	(8.4%)	1541	133	(8.6%)	454	32	(7.0%)	332	28	(8.4%)	73	8	(11.0%)
6 months	*N*	*n*	(%)	2325	116	(5.0%)	1494	82	(5.5%)	430	16	(3.7%)	328	14	(4.3%)	73	4	(5.5%)
De novo	*N*	*n*	(%)	2166	55	(2.5%)	1382	40	(2.9%)	408	8	(2.0%)	306	5	(1.6%)	70	2	(2.9%)
12 months	*N*	*n*	(%)	2370	125	(5.3%)	1520	84	(5.5%)	443	22	(5.0%)	334	16	(4.8%)	73	3	(4.1%)
De novo	*N*	*n*	(%)	2201	57	(2.6%)	1396	38	(2.7%)	422	11	(2.6%)	313	5	(1.6%)	70	3	(4.3%)
24 months	*N*	*n*	(%)	2070	112	(5.4%)	1324	78	(5.9%)	388	18	(4.6%)	292	14	(4.8%)	66	2	(3.0%)
De novo	*N*	*n*	(%)	1921	57	(3.0%)	1221	40	(3.3%)	365	9	(2.5%)	273	8	(2.9%)	62	0	(0.0%)
Vaginal pain (‘all of the time’)																		
Baseline	*N*	*n*	(%)	2380	81	(3.4%)	1536	52	(3.4%)	442	14	(3.2%)	329	14	(4.3%)	73	1	(1.4%)
12 months	*N*	*n*	(%)	2143	26	(1.2%)	1364	17	(1.2%)	401	2	(0.5%)	311	5	(1.6%)	67	2	(3.0%)
De novo	*N*	*n*	(%)	1982	14	(0.7%)	1256	12	(1.0%)	375	1	(0.3%)	287	0	(0.0%)	64	1	(1.6%)
24 months	*N*	*n*	(%)	2050	22	(1.1%)	1317	13	(1.0%)	385	4	(1.0%)	284	2	(0.7%)	64	3	(4.7%)
De novo	*N*	*n*	(%)	1886	14	(0.7%)	1211	8	(0.7%)	352	3	(0.9%)	263	0	(0.0%)	60	3	(5.0%)
Vaginal tightness (‘all of the time’)																		
Baseline	*N*	*n*	(%)	2273	13	(0.6%)	1462	11	(0.8%)	425	1	(0.2%)	319	1	(0.3%)	67	0	(0.0%)
12 months	*N*	*n*	(%)	2096	37	(1.8%)	1340	24	(1.8%)	387	5	(1.3%)	303	6	(2.0%)	66	2	(3.0%)
De novo	*N*	*n*	(%)	1868	31	(1.7%)	1184	19	(1.6%)	351	4	(1.1%)	273	6	(2.2%)	60	2	(3.3%)
24 months	*N*	*n*	(%)	1999	27	(1.4%)	1283	20	(1.6%)	376	2	(0.5%)	280	3	(1.1%)	60	2	(3.3%)
De novo	*N*	*n*	(%)	1777	24	(1.4%)	1134	18	(1.6%)	332	1	(0.3%)	256	3	(1.2%)	55	2	(3.6%)
Dyspareunia (‘a lot’)																		
Baseline	*N*	*n*	(%)	1211	116	(9.6%)	765	77	(10.1%)	229	18	(7.9%)	183	18	(9.8%)	34	3	(8.8%)
12 months	*N*	*n*	(%)	1059	58	(5.5%)	672	36	(5.4%)	201	14	(7.0%)	161	6	(3.7%)	25	2	(8.0%)
De novo	*N*	*n*	(%)	822	31	(3.8%)	515	18	(3.5%)	154	7	(4.5%)	132	5	(3.8%)	21	1	(4.8%)
24 months	*N*	*n*	(%)	948	44	(4.6%)	602	29	(4.8%)	179	6	(3.4%)	144	6	(4.2%)	23	3	(13.0%)
De novo	*N*	*n*	(%)	738	23	(3.1%)	462	16	(3.5%)	140	2	(1.4%)	115	4	(3.5%)	21	1	(4.8%)

Severe vaginal pain, before surgery, was reported by 3.4% (95% CI 2.7–4.2%) of women across all groups. The incidence of pain in the mesh kit group at baseline was lower than in other groups at 1.4% (95% CI 0.0–7.4%).

Severe dyspareunia, at baseline, was reported by 9.6% (95% CI 8.0–11.4%) of women.

At 2 years following surgery, there was an overall improvement in the rate of severe dyspareunia in all groups expect those who had a mesh kit. In this group, there was no change in the number of women who had severe dyspareunia. Vaginal pain also improved in all groups except in women who had a mesh kit, although the numbers in this group were low.

De novo vaginal tightness was more common after native tissue repair or biological xenograft than after a mesh inlay.

At baseline, using EQ‐5D‐3L, 8.4% (95% CI 7.3–9.6%) of women reported extreme pain or discomfort for any reason; this was not necessarily related to prolapse. This was found to have a sustained improvement after surgery across all methods of surgery (Table 4). However, at 24 months, extreme de novo generic pain developed in 3.0% (95% CI 2.3–3.8%) of women in all groups except the mesh kit group, in which there were no cases.

Rates of pain related to prolapse surgery, assessed using the IUGA/ICS classification, were low and results were similar in each group (Table S2).

## Discussion

### Main findings

Our study demonstrates the safety of both primary and secondary prolapse surgery but highlights areas of morbidity that are common, such as infection, urinary retention and vaginal adhesions. These could be highlighted in the patient information used in counselling before surgery and management could be improved by using evidence‐based protocols.

One of the most common complications was vaginal adhesions, occurring in 1 in 60 cases overall and 1 in 30 cases associated with a biological xenograft. Adhesions might reduce the risk of recurrent prolapse but may increase dyspareunia rates and may even result in women no longer being able to have penetrative sexual intercourse following their surgery. Further research should assess the use of estrogen on the formation of adhesions or the use of a vaginal insert to prevent adhesion formation.

Our study provides an important insight into the background incidence of concurrent pain with prolapse and the risk of developing de novo pain or dyspareunia following all types of prolapse surgery. It demonstrates the complexity of assessing pain and the need for both comparisons to baseline and other treatment options.

### Strengths

The key strengths of the PROSPECT study are its size, prospective data collection, inclusion of patient‐reported outcome measures and free‐text questions, its generalisability to current UK practice and the ability to describe complications of native tissue, biological xenograft and mesh repairs at 2 years. The inclusion of the large comprehensive cohort increases the generalisability and allows for much greater precision in the event rate estimates.

Complications may be under‐reported in routine clinical practice or registries. In our study, each hospital was asked to report any serious adverse event that occurred during the procedure to the point of discharge and the women were asked to report serious adverse events in their postal questionnaires, which were then confirmed with the hospital where possible; this dual method of reporting serious adverse events reduced but did not eliminate the possibility that complications could be under‐reported.

### Limitations

One possible limitation of the study is that randomised women were only examined once in the postoperative period at 12 months, which skewed the time point data for detection of asymptomatic mesh exposure. However, it would be expected that if women were symptomatic, they would have sought help.^15^

A limitation of this analysis is that any comparison between treatment groups must be made with caution. Given the definition of the groups by treatment received (rather than by randomised allocation), the combination of primary and secondary repairs and the inclusion of the non‐randomised cohort, comparisons could be subject to potentially significant confounding For this reason, we have sought only to present a descriptive summary of complication rates for each type of surgery rather than to make formal comparisons between treatments. A further statistical limitation arises from missing data, which are quite plausibly not missing at random. Although there was rigorous adherence to adverse event reporting and high questionnaire response rates (91% at 1 year and 80% at 2 years), there remains a risk of a small amount of bias in the results and the direction of this effect is difficult to determine. However, sensitivity analyses undertaken in the main trial under varying assumptions of missingness did not indicate non‐response bias.^5^

Different types of pain (generic, dyspareunia, vaginal) were assessed using several measures, PROMs, EQ‐5D‐3L and free‐text communications from women. The IUGA/ICS category time site (CTS) subclassification of provoked pain (a–‐e) was determined using women’s free‐text responses, so of the 241 women who complained of pain, only 46% could be categorised a to e.

Even in a study the size of PROSPECT, of 2632 women, serious rare complications might not be detected, which highlights the need for registries, such as the Medicines and Health Regulatory Agency, to which serious complications can be reported.

There was a relatively small number of surgeries performed with polypropylene mesh kits (*n* = 78; 3% of overall sample). These were mainly performed in cases of recurrent prolapse hence the numbers were much lower than other methods of surgery. Data from kits and inlays were presented separately because we believe that they represent significantly different operations. Inlays and kits were investigated in separate trials within PROSPECT for primary and secondary prolapse repairs, respectively. The study was designed to have sufficient statistical power in the primary trial, whereas the smaller trial of recurrent prolapse was exploratory.

Prolapse is a long‐term condition and there is a need to report complications for longer than 12 months. The follow‐up data from our study were collected for 2 years and ongoing 6 years follow‐up data are being collected.

### Interpretation in light of other evidence

The Cumberlege report called for further studies to inform women and clinicians of the risk of complications of mesh. Our study gives this and also provides the important contextual comparison to the risks of native tissue surgery. The pragmatic study design enables clinicians to use reliable data to counsel women about all the risks of prolapse surgery and also to benchmark their practice.

Initial studies of complications of transvaginal polypropylene mesh surgery for POP focused on mesh perforation, exposure and extrusion rates (‘erosion’).^16^ These can cause severe morbidity; however, they are relatively rare but in the absence of a registry it is impossible to accurately calculate their true incidence.

The British Society of Urogynaecology (BSUG) surgical audit database, has been used for 13 years and contains large amounts of data; nonetheless, not all procedures are included because reporting is not mandatory and is restricted to members of BSUG. Therefore, the recommendation by Cumberlege of mandatory reporting to a national registry is welcomed.

The quality of evidence concerning the risk of complications following polypropylene mesh surgery for both incontinence and prolapse remains poor. For example, research which does not have a comparative standard ‘native tissue’ group can present a biased evidence base whereas studies that only present data using hospital episode statistics data are limited by the type of clinical information available and potential coding errors.^17^ Another historical problem has been an over‐reliance on case series conducted in one expert unit, which are later found to not be generalisable.^18^, ^19^

The Cumberlege report called for greater knowledge of the incidence and the reasons for of loss of sex life, chronic pain, infection, difficulty voiding, de novo urinary incontinence, haemorrhage and damage to surrounding organs following mesh surgery and autoimmune diseases or psychiatric injury. This study provides most of this information not only for mesh surgery but importantly for all types of prolapse surgery (with the exception of autoimmune diseases or psychiatric injury).

This is the first study to report complications following mesh, biological xenograft and native tissue surgery using the IUGA/ICS classification code. Most reports in the literature are case series of mesh complications that report no denominator and no comparative group. More recent concerns have been raised about the incidence of pain and dyspareunia, both of which can have a delayed onset.^3^ Miklos et al.^3^ reported a cases series of 250 women. They found that 48.4% had a chief complaint of pain, 43.2% had vaginal exposure and only 4.8% had experienced perforation of an organ. Over 85% were reported more than 12 months after the index operation.

Miklos et al.^3^ stressed the importance of pain as a symptom. They felt that significantly more women were bothered by pain rather than mesh exposure. However, they did not report a control group and the denominator for these complication cases was unknown. Our study has demonstrated the complexity of assessing pain and the need to consider de novo pain. It suggests that severe de novo generic pain can occur in up to 3.0% of women 24 months after surgery but that it can occur after all types of prolapse surgery. The incidence of pain recorded from patient free‐text questionnaires, converted to the CTS classification was higher than that recorded using PROMs; this is possibly because severity was not considered. Also, the IUGA/ICS classification does not consider de novo pain.

One of the most difficult problems that clinicians currently face is counselling women about the risks and benefits of mesh removal surgery for pain, in the absence of exposure or extrusion (‘erosion’)/perforation. Our study does not help to answer this important question, but it demonstrates similar rates of severe de novo pain and dyspareunia after each type of prolapse surgery.

## Conclusion

This is the first study to prospectively address the complications of vaginal polypropylene mesh used for prolapse surgery alongside comparable data from both biological collagen xenographs and ‘standard’ native tissue repairs. It demonstrates that all types of prolapse surgery have low surgical morbidity and a low rate of severe complications. However, there are some women who will experience severe de novo pain, dyspareunia and other life‐changing morbidities after all types of prolapse surgery. Our results should help clinicians when counselling women who are contemplating a surgical treatment for their prolapse. Further research is required to standardise outcomes and to determine the best methods to treat complications.

### Disclosure of interests

Dr. Reid has nothing to disclose. Completed disclosure of interests form available to view online as supporting information.

### Contribution to authorship

FR, AE, SB and RF wrote the first draft of the manuscript which was reviewed, modified and approved by all authors. AE analysed the data, which were interpreted by all other authors. SB managed the PROSPECT study with support, input and oversight from FR and RF. All the authors vouch for the accuracy and completeness of the data reported.

### Details of ethics approval

PROSPECT was approved by the North of Scotland Research Ethics Committee (NOSRES) on 7 July 2009 (REC reference number 09/SO802/56).

### Funding

The project was funded by the National Institute for Health Research Health Technology Assessment Programme (Project Number 07/60/18). The Health Services Research Unit is funded by the Chief Scientist Office of the Scottish Government Health and Social Care Directorates.

### Acknowledgements

The authors wish to thank the women who participated in the PROSPECT study. We also thank Margaret MacNeil for her secretarial support and data management, the programming team in CHaRT and the staff at the recruitment sites who facilitated the recruitment, treatment and follow up of study participants.^6^

### Disclaimer

The views and opinions expressed herein are those of the authors and do not necessarily reflect those of the Health Technology Assessment Programme, the National Institute of Health Research, the National Health Service or the Department of Health.

## Supporting information


**Figure S1**. Radar Plots of IUGA/ICS classification of complications related directly to female pelvic reconstructive surgery.

**Table S1**. Complication rates by IUGA classification category and subcategory.
**Table S2**. IUGA classification of complications related to primary and secondary repairs.
**Table S3**. Complication rates by IUGA classification category and pain subclassification.

Supplementary Material

## Data Availability

Author elects to not share data.

## Appendix A

PROSPECT study group: CMA Glazener: Centre for Healthcare Randomised Trial, Health Services Research Unit, University of Aberdeen, Aberdeen, UK; C Hemming: Department of Obstetrics and Gynaecology, Aberdeen Royal Infirmary, Aberdeen, UK; KG Cooper: Department of Obstetrics and Gynaecology, Aberdeen Royal Infirmary, Aberdeen, UK, ARB Smith: The Warrell Unit, Saint Mary’s Hospital, Manchester University NHS Foundation Trust, Manchester Academic Health Science Centre, Hathersage Road, Manchester M13 9WL, UK and Institute of Human Development, Faculty of Medical & Human Sciences, University of Manchester, Manchester Academic Health Science Centre, Oxford Road, Manchester M13 9PL, UK; S Hagen: NMAHP Research Unit, Glasgow Caledonian University, Glasgow, UK; I Montgomery: Centre for Healthcare Randomised Trial, Health Services Research Unit, University of Aberdeen, Aberdeen, UK; M Kilonzo: Health Economics Research Unit, University of Aberdeen, Aberdeen, UK and Usher Institute of Population Health Sciences & Informatics, University of Edinburgh, Edinburgh, UK, D Boyers: Health Economics Research Unit, University of Aberdeen, Aberdeen, UK and Usher Institute of Population Health Sciences & Informatics, University of Edinburgh, Edinburgh, UK; A McDonald: Centre for Healthcare Randomised Trial, Health Services Research Unit, University of Aberdeen, Aberdeen, UK; G McPherson: Centre for Healthcare Randomised Trial, Health Services Research Unit, University of Aberdeen, Aberdeen, UK; G MacLennan: Centre for Healthcare Randomised Trial, Health Services Research Unit, University of Aberdeen, Aberdeen, UK, J Norrie: Health Economics Research Unit, University of Aberdeen, Aberdeen, UK and Usher Institute of Population Health Sciences & Informatics, University of Edinburgh, Edinburgh, UK.
