# Supplementary material for: How common are complications following polypropylene mesh, biological xenograft and native tissue surgery for pelvic organ prolapse? A secondary analysis from the PROSPECT trial
Source: BJOG. 2021 Sep 27;128(13):2180–9. doi: 10.1111/1471-0528.16897 (PMC9292877; doi:10.1111/1471-0528.16897)
Supplement: Supplementary file 1 — Figure S1. Radar Plots of IUGA/ICS classification of complications related directly to female pelvic reconstructive surgery. [file BJO-128-2180-s003.docx]

**Figure S1.** Radar Plots of IUGA/ICS classification of complications related directly female pelvic reconstructive surgery


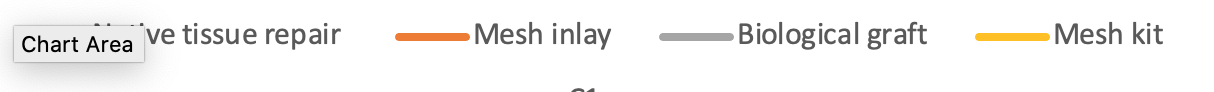


C1:vaginal: no epithelial separation;C2: vaginal: smaller ≤1 cm exposure; C3: vaginal: larger >1 cm exposure, or any extrusion;

C4: urinary tract: compromise or perforation including prosthesis (graft) perforation and fistula; C5: rectal or bowel: compromise or

perforation including prosthesis (graft) perforation and fistula; C6: skin or musculoskeletal:complications including discharge, pain, lump, or sinus tract formation; C7: patient: compromise including hematoma or systemic compromise.

TIME : T1 Intraoperative to 48 h T2 48 h to 2 months , T3 2months to 12months T4 Over 12 months

SITE S1:vaginal: area of the suture line;S2: vaginal: away from the suture line; S3: trocar passage; S4: other skin or musculoskeletal site; S5: intra-abdominal.

PAIN : U: unspecified; a: asymptomatic or no pain; b: provoked pain only; c: pain during sexual intercourse; d: pain during physical activities; e: spontaneous pain
