# Supplementary material for: How common are complications following polypropylene mesh, biological xenograft and native tissue surgery for pelvic organ prolapse? A secondary analysis from the PROSPECT trial
Source: BJOG. 2021 Sep 27;128(13):2180–9. doi: 10.1111/1471-0528.16897 (PMC9292877; doi:10.1111/1471-0528.16897)
Supplement: Supplementary file 2 — Table S1. Complication rates by IUGA classification category and subcategory. Table S2. IUGA classification of complications related to primary and secondary repairs. Table S3. Complication rates by IUGA classification category and pain subclassification. [file BJO-128-2180-s002.docx]

**Table S1.** Complication rates by IUGA classification category and sub-category

|  | | **All procedures** | | **Native tissue repair** | | **Mesh inlay** | | **Biological xenograft** | | **Mesh kit** | |
| --- | --- | --- | --- | --- | --- | --- | --- | --- | --- | --- | --- |
|  |  | **N = 2632** | | **N = 1712** | | **N = 482** | | **N = 360** | | **N = 78** | |
| 1. Vaginal: no epithelial separation | n (%) | 178 | 6.8% | 104 | 6.1% | 30 | 6.2% | 37 | 10.3% | 7 | 9.0% |
| —1A: Abnormal finding, prosthesis or graft on clinical examination | n (%) | 36 | 1.4% | 16 | 0.9% | 9 | 1.9% | 10 | 2.8% | 1 | 1.3% |
| —1B: Symptomatic e.g. unusual discomfort/pain, dyspareunia, bleeding | n (%) | 87 | 3.3% | 53 | 3.1% | 13 | 2.7% | 16 | 4.4% | 5 | 6.4% |
| —1C/1D: Infection (suspected or actual)/Abscess | n (%) | 64 | 2.4% | 41 | 2.4% | 9 | 1.9% | 13 | 3.6% | 1 | 1.3% |
| 2. Vaginal: smaller <=1cm exposure | n (%) | 49 | 1.9% | 3 | 0.2% | 39 | 8.1% | 1 | 0.3% | 6 | 7.7% |
| —2A: Asymptomatic | n (%) | 32 | 1.2% | 3 | 0.2% | 26 | 5.4% | 1 | 0.3% | 2 | 2.6% |
| —2B: Symptomatic | n (%) | 18 | 0.7% | 0 | 0.0% | 14 | 2.9% | 0 | 0.0% | 4 | 5.1% |
| —2C/2D: Infection/Abscess | n (%) | 0 | 0.0% | 0 | 0.0% | 0 | 0.0% | 0 | 0.0% | 0 | 0.0% |
| 3. Vaginal: larger >1cm exposure, or any extrusion | n (%) | 24 | 0.9% | 2 | 0.1% | 21 | 4.4% | 0 | 0.0% | 1 | 1.3% |
| —3A: Asymptomatic | n (%) | 17 | 0.6% | 2 | 0.1% | 15 | 3.1% | 0 | 0.0% | 0 | 0.0% |
| —3B: Symptomatic | n (%) | 7 | 0.3% | 0 | 0.0% | 7 | 1.5% | 0 | 0.0% | 0 | 0.0% |
| —3C/3D: Infection/Abscess | n (%) | 1 | 0.0% | 0 | 0.0% | 0 | 0.0% | 0 | 0.0% | 1 | 1.3% |
| 4. Urinary tract: compromise or perforation | n (%) | 84 | 3.2% | 57 | 3.3% | 13 | 2.7% | 10 | 2.8% | 4 | 5.1% |
| —4A: Small intraoperative defect | n (%) | 10 | 0.4% | 9 | 0.5% | 1 | 0.2% | 0 | 0.0% | 0 | 0.0% |
| —4B: Other lower urinary tract complication or urinary retention | n (%) | 75 | 2.8% | 50 | 2.9% | 11 | 2.3% | 10 | 2.8% | 4 | 5.1% |
| —4C: Ureteric or upper urinary tract complication | n (%) | 1 | 0.0% | 0 | 0.0% | 1 | 0.2% | 0 | 0.0% | 0 | 0.0% |
| 5. Rectal or bowel: compromise or perforation | n (%) | 5 | 0.2% | 1 | 0.1% | 3 | 0.6% | 1 | 0.3% | 0 | 0.0% |
| —5A: Small intraoperative defect | n (%) | 1 | 0.0% | 0 | 0.0% | 0 | 0.0% | 1 | 0.3% | 0 | 0.0% |
| —5B: Rectal injury or compromise | n (%) | 3 | 0.1% | 1 | 0.1% | 2 | 0.4% | 0 | 0.0% | 0 | 0.0% |
| —5C/5D: Bowel injury, compromise or abscess | n (%) | 1 | 0.0% | 0 | 0.0% | 1 | 0.2% | 0 | 0.0% | 0 | 0.0% |
| 6. Skin or musculoskeletal: complications | n (%) | 15 | 0.6% | 8 | 0.5% | 6 | 1.2% | 1 | 0.3% | 0 | 0.0% |
| —6A: Asymptomatic, abnormal finding on clinical examination | n (%) | 0 | 0.0% | 0 | 0.0% | 0 | 0.0% | 0 | 0.0% | 0 | 0.0% |
| —6B: Symptomatic e.g. discharge, pain or lump | n (%) | 13 | 0.5% | 6 | 0.4% | 6 | 1.2% | 1 | 0.3% | 0 | 0.0% |
| —6C/6D: Infection (e.g. sinus tract formation)/Abscess | n (%) | 2 | 0.1% | 2 | 0.1% | 0 | 0.0% | 0 | 0.0% | 0 | 0.0% |
| 7. Patient: compromise | n (%) | 67 | 2.5% | 39 | 2.3% | 15 | 3.1% | 10 | 2.8% | 3 | 3.8% |
| —7A: Bleeding complication including haematoma | n (%) | 57 | 2.2% | 33 | 1.9% | 13 | 2.7% | 9 | 2.5% | 2 | 2.6% |
| —7B: Major degree of resuscitation or intensive care | n (%) | 11 | 0.4% | 7 | 0.4% | 2 | 0.4% | 1 | 0.3% | 1 | 1.3% |
| —7C: Mortality | n (%) | 0 | 0.0% | 0 | 0.0% | 0 | 0.0% | 0 | 0.0% | 0 | 0.0% |

**Table S2.** IUGA classification of complications related to primary and secondary repairs

|  | | **All procedures** | | | | **Native tissue repair** | | | | **Mesh inlay** | | | | **Biological xenograft** | | | | **Mesh kit** | | | |
| --- | --- | --- | --- | --- | --- | --- | --- | --- | --- | --- | --- | --- | --- | --- | --- | --- | --- | --- | --- | --- | --- |
|  |  | **Primary** | | **Secondary** | | **Primary** | | **Secondary** | | **Primary** | | **Secondary** | | **Primary** | | **Secondary** | | **Primary** | | **Secondary** | |
|  |  | **N = 2275** | | **N = 357** | | **N = 1527** | | **N = 185** | | **N = 387** | | **N = 95** | | **N = 341** | | **N = 19** | | **N = 20** | | **N = 58** | |
| **General Description** | | | | | | | | | | | | | | | | | | | | | |
| —1. Vaginal: no epithelial separation | n (%) | 152 | (6.7%) | 26 | (7.3%) | 91 | (6.0%) | 13 | (7.0%) | 25 | (6.5%) | 5 | (5.3%) | 35 | (10.3%) | 2 | (10.5%) | 1 | (5.0%) | 6 | (10.3%) |
| —2. Vaginal: smaller <=1cm exposure | n (%) | 37 | (1.6%) | 12 | (3.4%) | 3 | (0.2%) | 0 | (0.0%) | 32 | (8.3%) | 7 | (7.4%) | 1 | (0.3%) | 0 | (0.0%) | 1 | (5.0%) | 5 | (8.6%) |
| —3. Vaginal: larger >1cm exposure, or any extrusion | n (%) | 20 | (0.9%) | 4 | (1.1%) | 2 | (0.1%) | 0 | (0.0%) | 17 | (4.4%) | 4 | (4.2%) | 0 | (0.0%) | 0 | (0.0%) | 1 | (5.0%) | 0 | (0.0%) |
| —4. Urinary tract: compromise or perforation | n (%) | 73 | (3.2%) | 11 | (3.1%) | 51 | (3.3%) | 6 | (3.2%) | 11 | (2.8%) | 2 | (2.1%) | 10 | (2.9%) | 0 | (0.0%) | 1 | (5.0%) | 3 | (5.2%) |
| —5. Rectal or bowel: compromise or perforation | n (%) | 4 | (0.2%) | 1 | (0.3%) | 1 | (0.1%) | 0 | (0.0%) | 2 | (0.5%) | 1 | (1.1%) | 1 | (0.3%) | 0 | (0.0%) | 0 | (0.0%) | 0 | (0.0%) |
| —6. Skin or musculoskeletal: complications | n (%) | 12 | (0.5%) | 3 | (0.8%) | 6 | (0.4%) | 2 | (1.1%) | 5 | (1.3%) | 1 | (1.1%) | 1 | (0.3%) | 0 | (0.0%) | 0 | (0.0%) | 0 | (0.0%) |
| —7. Patient: compromise | n (%) | 57 | (2.5%) | 10 | (2.8%) | 36 | (2.4%) | 3 | (1.6%) | 12 | (3.1%) | 3 | (3.2%) | 9 | (2.6%) | 1 | (5.3%) | 0 | (0.0%) | 3 | (5.2%) |
| **Time (clinically diagnosed)** | | | | | | | | | | | | | | | | | | | | | |
| —T1: Intraoperative to 48 h | n (%) | 7 | (0.3%) | 2 | (0.6%) | 5 | (0.3%) | 2 | (1.1%) | 2 | (0.5%) | 0 | (0.0%) | 0 | (0.0%) | 0 | (0.0%) | 0 | (0.0%) | 0 | (0.0%) |
| —T2: 48 h to 2 months | n (%) | 7 | (0.3%) | 0 | (0.0%) | 6 | (0.4%) | 0 | (0.0%) | 0 | (0.0%) | 0 | (0.0%) | 1 | (0.3%) | 0 | (0.0%) | 0 | (0.0%) | 0 | (0.0%) |
| —T3: 2 months to 12 months | n (%) | 273 | (12.0%) | 43 | (12.0%) | 154 | (10.1%) | 18 | (9.7%) | 72 | (18.6%) | 13 | (13.7%) | 44 | (12.9%) | 2 | (10.5%) | 3 | (15.0%) | 10 | (17.2%) |
| —T4: over 12 months | n (%) | 46 | (2.0%) | 17 | (4.8%) | 15 | (1.0%) | 3 | (1.6%) | 22 | (5.7%) | 8 | (8.4%) | 9 | (2.6%) | 2 | (10.5%) | 0 | (0.0%) | 4 | (6.9%) |
| **Site** | | | | | | | | | | | | | | | | | | | | | |
| —S1: vaginal: area of suture line | n (%) | 90 | (4.0%) | 14 | (3.9%) | 32 | (2.1%) | 2 | (1.1%) | 39 | (10.1%) | 9 | (9.5%) | 18 | (5.3%) | 1 | (5.3%) | 1 | (5.0%) | 2 | (3.4%) |
| —S2: vaginal: away from suture line | n (%) | 134 | (5.9%) | 34 | (9.5%) | 75 | (4.9%) | 13 | (7.0%) | 33 | (8.5%) | 10 | (10.5%) | 24 | (7.0%) | 1 | (5.3%) | 2 | (10.0%) | 10 | (17.2%) |
| —S3: adjoining viscus / trocar passage * | n (%) | 85 | (3.7%) | 13 | (3.6%) | 59 | (3.9%) | 7 | (3.8%) | 14 | (3.6%) | 3 | (3.2%) | 11 | (3.2%) | 0 | (0.0%) | 1 | (5.0%) | 3 | (5.2%) |
| —S4: other skin or musculoskeletal site | n (%) | 14 | (0.6%) | 3 | (0.8%) | 8 | (0.5%) | 2 | (1.1%) | 5 | (1.3%) | 1 | (1.1%) | 1 | (0.3%) | 0 | (0.0%) | 0 | (0.0%) | 0 | (0.0%) |
| —S5: intra-abdominal | n (%) | 17 | (0.7%) | 0 | (0.0%) | 12 | (0.8%) | 0 | (0.0%) | 3 | (0.8%) | 0 | (0.0%) | 2 | (0.6%) | 0 | (0.0%) | 0 | (0.0%) | 0 | (0.0%) |
| **Pain** | | | | | | | | | | | | | | | | | | | | | |
| —U: unspecified | n (%) | 111 | (4.9%) | 20 | (5.6%) | 60 | (3.9%) | 7 | (3.8%) | 28 | (7.2%) | 7 | (7.4%) | 21 | (6.2%) | 0 | (0.0%) | 2 | (10.0%) | 6 | (10.3%) |
| —a: asymptomatic or no pain | n (%) | 129 | (5.7%) | 15 | (4.2%) | 68 | (4.5%) | 4 | (2.2%) | 40 | (10.3%) | 6 | (6.3%) | 20 | (5.9%) | 1 | (5.3%) | 1 | (5.0%) | 4 | (6.9%) |
| —b: provoked pain only | n (%) | 5 | (0.2%) | 3 | (0.8%) | 3 | (0.2%) | 0 | (0.0%) | 1 | (0.3%) | 1 | (1.1%) | 1 | (0.3%) | 1 | (5.3%) | 0 | (0.0%) | 1 | (1.7%) |
| —c: pain during sexual intercourse | n (%) | 29 | (1.3%) | 4 | (1.1%) | 18 | (1.2%) | 2 | (1.1%) | 6 | (1.6%) | 2 | (2.1%) | 5 | (1.5%) | 0 | (0.0%) | 0 | (0.0%) | 0 | (0.0%) |
| —d: pain during physical activities | n (%) | 4 | (0.2%) | 0 | (0.0%) | 3 | (0.2%) | 0 | (0.0%) | 1 | (0.3%) | 0 | (0.0%) | 0 | (0.0%) | 0 | (0.0%) | 0 | (0.0%) | 0 | (0.0%) |
| —e: spontaneous pain | n (%) | 48 | (2.1%) | 17 | (4.8%) | 24 | (1.6%) | 10 | (5.4%) | 17 | (4.4%) | 5 | (5.3%) | 7 | (2.1%) | 0 | (0.0%) | 0 | (0.0%) | 2 | (3.4%) |

*Adjoining viscus (e.g. bladder or bowel) for native tissue repairs and trocar passage for mesh and repairs.

**Table S3.** Complication rates by IUGA classification category and pain sub-classification

|  | | **All procedures** | | **Native tissue repair** | | **Mesh inlay** | | **Biological xenograft** | | **Mesh kit** | |
| --- | --- | --- | --- | --- | --- | --- | --- | --- | --- | --- | --- |
|  |  | **N = 2632** | | **N = 1712** | | **N = 482** | | **N = 360** | | **N = 78** | |
| 1. Vaginal: no epithelial separation | n (%) | 178 | 6.8% | 104 | 6.1% | 30 | 6.2% | 37 | 10.3% | 7 | 9.0% |
| —a: asymptomatic or no surgery related pain | n (%) | 51 | 1.9% | 23 | 1.3% | 13 | 2.7% | 14 | 3.9% | 1 | 1.3% |
| —b: provoked pain only | n (%) | 7 | 0.3% | 3 | 0.2% | 1 | 0.2% | 2 | 0.6% | 1 | 1.3% |
| —c: pain during sexual intercourse | n (%) | 30 | 1.1% | 20 | 1.2% | 5 | 1.0% | 5 | 1.4% | 0 | 0.0% |
| —d: pain during physical activities | n (%) | 2 | 0.1% | 2 | 0.1% | 0 | 0.0% | 0 | 0.0% | 0 | 0.0% |
| —e: spontaneous pain | n (%) | 36 | 1.4% | 23 | 1.3% | 8 | 1.7% | 5 | 1.4% | 0 | 0.0% |
| —pain not specified | n (%) | 58 | 2.2% | 35 | 2.0% | 4 | 0.8% | 14 | 3.9% | 5 | 6.4% |
| 2. Vaginal: smaller <=1cm exposure | n (%) | 49 | 1.9% | 3 | 0.2% | 39 | 8.1% | 1 | 0.3% | 6 | 7.7% |
| —a: asymptomatic or no surgery related pain | n (%) | 19 | 0.7% | 2 | 0.1% | 15 | 3.1% | 1 | 0.3% | 1 | 1.3% |
| —b: provoked pain only | n (%) | 0 | 0.0% | 0 | 0.0% | 0 | 0.0% | 0 | 0.0% | 0 | 0.0% |
| —c: pain during sexual intercourse | n (%) | 1 | 0.0% | 0 | 0.0% | 1 | 0.2% | 0 | 0.0% | 0 | 0.0% |
| —d: pain during physical activities | n (%) | 0 | 0.0% | 0 | 0.0% | 0 | 0.0% | 0 | 0.0% | 0 | 0.0% |
| —e: spontaneous pain | n (%) | 6 | 0.2% | 0 | 0.0% | 5 | 1.0% | 0 | 0.0% | 1 | 1.3% |
| —pain not specified | n (%) | 25 | 0.9% | 1 | 0.1% | 20 | 4.1% | 0 | 0.0% | 4 | 5.1% |
| 3. Vaginal: larger >1 cm exposure, or any extrusion | n (%) | 24 | 0.9% | 2 | 0.1% | 21 | 4.4% | 0 | 0.0% | 1 | 1.3% |
| —a: asymptomatic or no surgery related pain | n (%) | 9 | 0.3% | 2 | 0.1% | 6 | 1.2% | 0 | 0.0% | 1 | 1.3% |
| —b: provoked pain only | n (%) | 1 | 0.0% | 0 | 0.0% | 1 | 0.2% | 0 | 0.0% | 0 | 0.0% |
| —c: pain during sexual intercourse | n (%) | 2 | 0.1% | 0 | 0.0% | 2 | 0.4% | 0 | 0.0% | 0 | 0.0% |
| —d: pain during physical activities | n (%) | 0 | 0.0% | 0 | 0.0% | 0 | 0.0% | 0 | 0.0% | 0 | 0.0% |
| —e: spontaneous pain | n (%) | 2 | 0.1% | 0 | 0.0% | 2 | 0.4% | 0 | 0.0% | 0 | 0.0% |
| —pain not specified | n (%) | 11 | 0.4% | 0 | 0.0% | 11 | 2.3% | 0 | 0.0% | 0 | 0.0% |
| 4. Urinary tract: compromise or perforation | n (%) | 84 | 3.2% | 57 | 3.3% | 13 | 2.7% | 10 | 2.8% | 4 | 5.1% |
| —a: asymptomatic or no pain | n (%) | 37 | 1.4% | 28 | 1.6% | 5 | 1.0% | 3 | 0.8% | 1 | 1.3% |
| —b: provoked pain only | n (%) | 0 | 0.0% | 0 | 0.0% | 0 | 0.0% | 0 | 0.0% | 0 | 0.0% |
| —c: pain during sexual intercourse | n (%) | 0 | 0.0% | 0 | 0.0% | 0 | 0.0% | 0 | 0.0% | 0 | 0.0% |
| —d: pain during physical activities | n (%) | 0 | 0.0% | 0 | 0.0% | 0 | 0.0% | 0 | 0.0% | 0 | 0.0% |
| —e: spontaneous pain | n (%) | 6 | 0.2% | 4 | 0.2% | 1 | 0.2% | 1 | 0.3% | 0 | 0.0% |
| —pain not specified | n (%) | 42 | 1.6% | 26 | 1.5% | 7 | 1.5% | 6 | 1.7% | 3 | 3.8% |
| 5. Rectal or bowel: compromise or perforation | n (%) | 5 | 0.2% | 1 | 0.1% | 3 | 0.6% | 1 | 0.3% | 0 | 0.0% |
| —a: asymptomatic or no pain | n (%) | 3 | 0.1% | 1 | 0.1% | 2 | 0.4% | 0 | 0.0% | 0 | 0.0% |
| —b: provoked pain only | n (%) | 0 | 0.0% | 0 | 0.0% | 0 | 0.0% | 0 | 0.0% | 0 | 0.0% |
| —c: pain during sexual intercourse | n (%) | 0 | 0.0% | 0 | 0.0% | 0 | 0.0% | 0 | 0.0% | 0 | 0.0% |
| —d: pain during physical activities | n (%) | 0 | 0.0% | 0 | 0.0% | 0 | 0.0% | 0 | 0.0% | 0 | 0.0% |
| —e: spontaneous pain | n (%) | 1 | 0.0% | 0 | 0.0% | 1 | 0.2% | 0 | 0.0% | 0 | 0.0% |
| —pain not specified | n (%) | 1 | 0.0% | 0 | 0.0% | 0 | 0.0% | 1 | 0.3% | 0 | 0.0% |
| 6. Skin or musculoskeletal: complications | n (%) | 15 | 0.6% | 8 | 0.5% | 6 | 1.2% | 1 | 0.3% | 0 | 0.0% |
| —a: asymptomatic or no pain | n (%) | 0 | 0.0% | 0 | 0.0% | 0 | 0.0% | 0 | 0.0% | 0 | 0.0% |
| —b: provoked pain only | n (%) | 0 | 0.0% | 0 | 0.0% | 0 | 0.0% | 0 | 0.0% | 0 | 0.0% |
| —c: pain during sexual intercourse | n (%) | 0 | 0.0% | 0 | 0.0% | 0 | 0.0% | 0 | 0.0% | 0 | 0.0% |
| —d: pain during physical activities | n (%) | 2 | 0.1% | 1 | 0.1% | 1 | 0.2% | 0 | 0.0% | 0 | 0.0% |
| —e: spontaneous pain | n (%) | 7 | 0.3% | 2 | 0.1% | 4 | 0.8% | 1 | 0.3% | 0 | 0.0% |
| —pain not specified | n (%) | 6 | 0.2% | 5 | 0.3% | 1 | 0.2% | 0 | 0.0% | 0 | 0.0% |
| 7. Patient: compromise | n (%) | 67 | 2.5% | 39 | 2.3% | 15 | 3.1% | 10 | 2.8% | 3 | 3.8% |
| —a: asymptomatic or no pain | n (%) | 36 | 1.4% | 19 | 1.1% | 10 | 2.1% | 6 | 1.7% | 1 | 1.3% |
| —b: provoked pain only | n (%) | 0 | 0.0% | 0 | 0.0% | 0 | 0.0% | 0 | 0.0% | 0 | 0.0% |
| —c: pain during sexual intercourse | n (%) | 0 | 0.0% | 0 | 0.0% | 0 | 0.0% | 0 | 0.0% | 0 | 0.0% |
| —d: pain during physical activities | n (%) | 0 | 0.0% | 0 | 0.0% | 0 | 0.0% | 0 | 0.0% | 0 | 0.0% |
| —e: spontaneous pain | n (%) | 11 | 0.4% | 7 | 0.4% | 3 | 0.6% | 0 | 0.0% | 1 | 1.3% |
| —pain not specified | n (%) | 21 | 0.8% | 14 | 0.8% | 2 | 0.4% | 4 | 1.1% | 1 | 1.3% |
